# Establishment of predictive nomogram and web-based survival risk calculator for malignant pleural mesothelioma: A SEER database analysis

**DOI:** 10.3389/fonc.2022.1027149

**Published:** 2022-10-07

**Authors:** Sihao Chen, Wanli Yu, Shilong Shao, Jie Xiao, Hansong Bai, Yu Pu, Mengxia Li

**Affiliations:** ^1^ Cancer Center, Daping Hospital, Army Medical University (Third Military Medical University), Chongqing, China; ^2^ Department of Neurosurgery, Chongqing General Hospital, University of Chinese Academy of Sciences, Chongqing, China; ^3^ Graduate Institute, Chongqing Medical University, Chongqing, China; ^4^ Department of Radiation Oncology, Sichuan Cancer Hospital & Institute, School of Medicine, University of Electronic Science and Technology of China, Sichuan Cancer Center, Radiation Oncology Key Laboratory of Sichuan Province, Chengdu, China

**Keywords:** malignant pleural mesothelioma (MPM), nomogram, prognosis, surveillance, epidemiology, and end results (SEER), risk-group classification

## Abstract

**Background:**

Malignant pleural mesothelioma (MPM) is an uncommon condition with limited available therapies and dismal prognoses. The purpose of this work was to create a multivariate clinical prognostic nomogram and a web-based survival risk calculator to forecast patients’ prognoses.

**Methods:**

Using a randomization process, training and validation groups were created for a retrospective cohort study that examined the Surveillance, Epidemiology, and End Results (SEER) database from 2010 to 2015 for individuals diagnosed with MPM (7:3 ratio). Overall survival (OS) and cancer-specific survival (CSS) were the primary endpoints. Clinical traits linked to OS and CSS were identified using Least Absolute Shrinkage and Selection Operator (LASSO) Cox regression analysis, which was also utilized to develop nomogram survival models and online survival risk calculators. By charting the receiver operating characteristic (ROC), consistency index (C-index), calibration curve, and decision curve analysis (DCA), the model’s performance was assessed. The nomogram was used to classify patients into various risk categories, and the Kaplan-Meier method was used to examine each risk group’s survival rate.

**Results:**

The prognostic model comprised a total of 1978 patients. For the total group, the median OS and CSS were 10 (9.4-10.5) and 11 (9.4-12.6) months, respectively. As independent factors for OS and CSS, age, gender, insurance, histology, T stage, M stage, surgery, and chemotherapy were chosen. The calibration graphs demonstrated good concordance. In the training and validation groups, the C-indices for OS and CSS were 0.729, 0.717, 0.711, and 0.721, respectively. Our nomogram produced a greater clinical net benefit than the AJCC 7th edition, according to DCA and ROC analysis. According to the cut-off values of 171 for OS and 189 for CSS of the total scores from our nomogram, patients were classified into two risk groups. The P-value < 0.001 on the Kaplan-Meier plot revealed a significant difference in survival between the two patient groups.

**Conclusions:**

Patient survival in MPM was correctly predicted by the risk evaluation model. This will support clinicians in the practice of individualized medicine.

## Introduction

Malignant pleural mesothelioma (MPM) is an uncommon form of pleural cancer with asbestos exposure as a risk factor (1, 2). The WHO predicts that asbestos-related illnesses, such as MPM, may become more common in the upcoming decades because asbestos has not been outlawed globally (3). MPM starts off slowly but becomes aggressively localized. The majority of patients are diagnosed at advanced stages, which are challenging to treat, and seldom recover (4, 5). Even patients with limited disease may suffer more acute shortness of breath and chest pain due to the fact that clinical symptoms are frequently more severe than those of other malignancies. The 5-year overall survival rate is less than 10%, and the median survival time is only approximately 1 year (6).

Due to the limited and passive nature of existing treatments for MPM, only minor improvements in survival rates and symptom relief have been observed until very recently (7). The National Comprehensive Cancer Network (NCCN) recommendations state that surgery, chemotherapy, and radiation therapy are the main current therapeutic options for MPM (8, 9). Patients with stage I-IIIA MPM who can have it surgically removed are given preference, while those with stage III-IV and unresectable stage I-IIIA are given pemetrexed combination with cisplatin chemotherapy as their initial treatment option (10, 11). Adjuvant radiation therapy is suggested for patients after radical resection to enhance local control in the management of MPM, however this is debatable (12). Numerous genomic investigations have demonstrated that targeted therapy is often ineffective because MPM proto-oncogene mutations are extremely uncommon (13). After platinum-based chemotherapy, dual immune checkpoint inhibitors (ICIs) therapy is the only first-line treatment approved for MPM based on the favorable results from CheckMate 743 study (14). The study found that combination of nivolumab and ipilimumab significantly decreased the risk of death by 26% in individuals with unresectable MPM when compared to conventional chemotherapy (pemetrexed+cisplatin). Patients in the dual ICIs group had a longer median OS of 18.1 months than those in the chemotherapy group, which had a median OS of 14.1 months (HR=0.74, 95% CI, 0.60-0.91, P=0.002).

Although the TNM staging system is widely reconginzed as standard protocol for prognosis evaluation, several studies have shown that the current TNM staging system for MPM patients is inadequate in regard of the correctness of N staging and individualized variability (15–18). In addition, the absence of a staging system with clinical and pathological features will also lead to dilemma in treatment algorism and prognostic assessment. In this study, we developed and validated a dynamic survival risk evaluation system based on large population data combined with existing clinical characteristics to bridge the gap in the current prognostic system.

## Methods

### Data source and selection criteria

The study adhered to the transparent reporting of a multivariable prediction model for individual prognosis or diagnosis (TRIPOD) reporting criteria for prognostic studies and was carried out in compliance with the Declaration of Helsinki (revised in 2013). The SEER database was used without institutional review board consent because it is a freely accessible resource. The SEER* Stat program (version 8.3.8; https://seer.cancer.gov/seerstat) was used to extract patient data from the updated SEER database (https://seer.cancer.gov).

**Figure 1:**
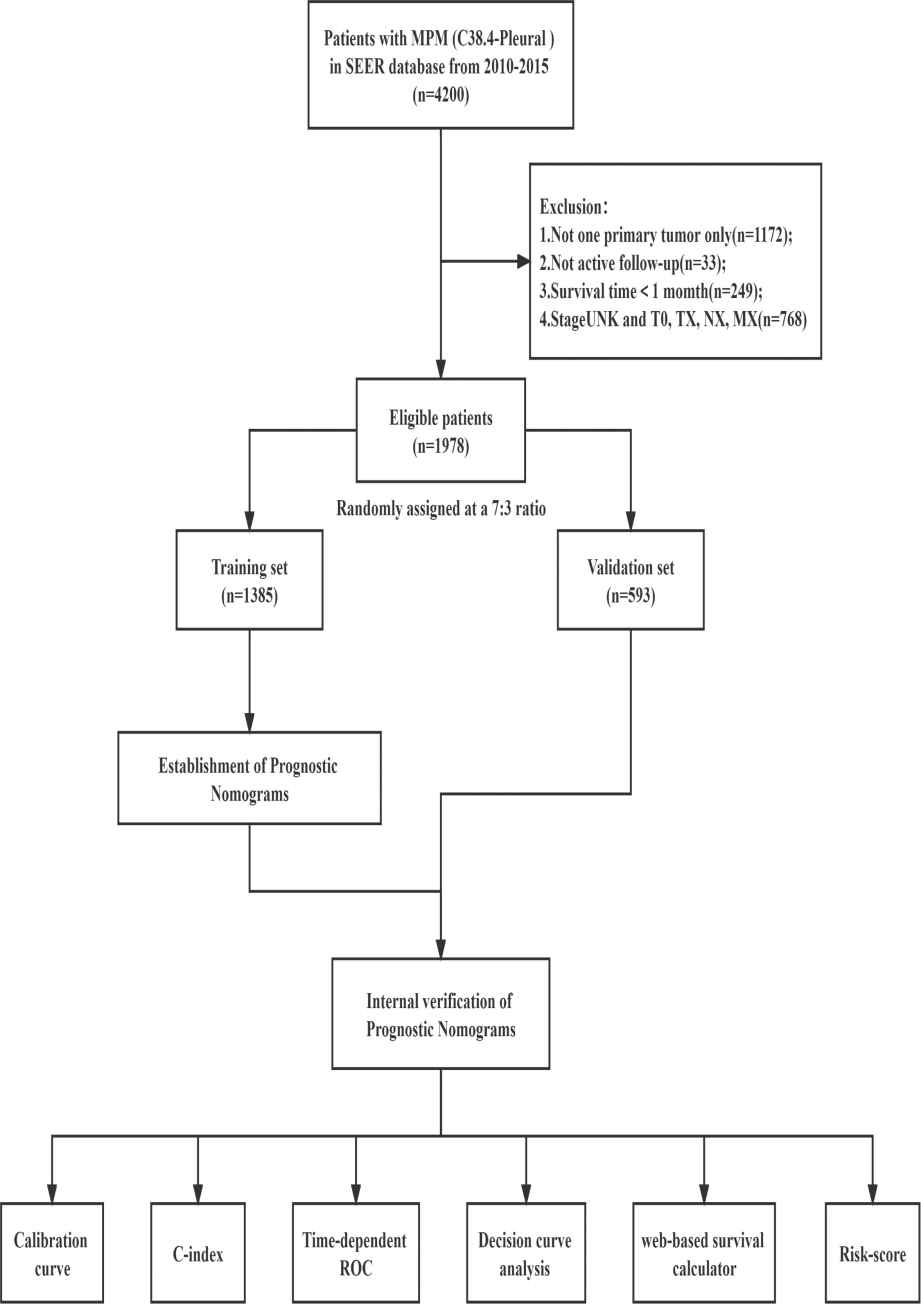
Study design and the workflow diagram.

The following were the screening standards (1): Patients with malignant pleural mesothelioma diagnosed between 2010 and 2015, coded as C38.4-Pleural and one primary tumor only, in accordance with the International Classification of Diseases for Oncology, Third Edition (ICD-O-3); (2) Patients with histopathological confirmation (coded as 9050-9053 in the ICD-O-3); (3) Patients with a known survival time and > 1 month; (4) Patients with full follow-up data; (5) Those having extensive information on characteristics such as vital status, survival months, age, sex, race, marital status, insurance, grade, site of laterality, 7th ed T/N/M/AJCC stage group, and primary tumor therapy modality (**Figure 1**). Overall survival (OS) was defined as the time from initial diagnosis of MPM to the last follow-up or death and calculated using the variables of “vital status”. Cancer-specific survival (CSS) was defined as the interval between the date of diagnosis of MPM and the date of death due to MPM and calculated using the variable of “SEER cause-specific death classification”. Treatment data were extracted from the following fields: radiation sequence with surgery, reason for no cancer-directed surgery, radiation recode, and chemotherapy recode. Patients were identified as having received cancer-directed surgery if given any of the following codes for the “Rx Summ-Surg Prim Site” variable: 30,40,50,60.

### Statistical analysis

The SPSS 26.0 and R software version 4.1.1 (https://www.r-project.org/) were used for all analyses, together with the rms, glment, survminer, timeROC, ggDCA, ggRisk, DynNom, and shine packages. The cutoff for statistical significance was P < 0.05. Data from the entire population that was taken from the SEER database was divided at random into training and validation groups in a 7:3 ratio. The chi-squared test was used for the analysis of all categorical variables, which were all provided as frequencies and percentages. The Kaplan-Meier method was used to create the survival curves, and the log-rank test was used to compare them. To find predictors linked to OS and CSS, a penalized Cox proportional hazards model employing the Adaptive Least Absolute Shrinkage and Selection Operator (LASSO) was applied to the development cohort, which effectively avoided over-fitting in the selection of significant features (19). The prognostic nomogram was then created using independent prognostic factors (P < 0.05), and it was used to plot the risk model on a web-based survival calculator.

Area under the time-dependent receiver operating characteristic curve (time-dependent ROC) and the concordance index (C-index) were used to measure discrimination, ranging from 0.5 to 1.0, representing the random probability of not discriminating to optimal discrimination (20). To assess the agreement between the anticipated survival probability and the observed probability, calibration curves were built. The predictive value of our model and the American Joint Committee on Cancer (AJCC) stage were compared using decision curve analysis (DCA) and time-dependent ROC. Unlike time-dependent ROC, DCA can further be used to determine the net clinical benefit of clinical outcomes at different probability thresholds (21). Individual risk scores were calculated according to the established nomograms. In order to further categorize patients into high- and low-risk groups, the best thresholds for OS and CSS were established using the Surv_Cutpoint function. Heatmaps of risk factor associations were used to illustrate the distribution of clinicopathological characteristics in different risk groups for OS and CSS.

## Results

### Characteristics of patients

There were a total of 1978 MPM patients in the entire cohort from the SEER database from 2010 to 2015 who met the inclusion criteria. After the patients were randomly divided into two groups in a ratio of 7:3, with 1385 and 593 patients in the training and validation groups, respectively, the patients were evaluated for their differences in clinical traits. Between the training and validation groups, there was no statistically significant variation in the patient distribution (P > 0.05). **Table 1** displays the demographic and clinical traits of the patients. Patients who were elderly (age>65, n=1454, 73.5%), men (n=1545, 78.1%), white people (n=1785, 90.2%), insured people (n=1472, 74.4%), and married people (n=1301, 65.8%) made up the majority of the patient population. About 63.2% of patients, according to the 7th edition of the AJCC staging system, were in advanced stages (stages III-IV). According to the therapeutic modalities used, 31.6% of patients underwent surgery, 59.0% used chemotherapy, and 13.5% underwent radiotherapy. The median OS and CSS times for the total population in the SEER database were 10 (9.5-10.5) months and 11 (9.4-12.6) months, respectively. Additionally, in the training group, the median OS and CSS were 10 (9.4-10.6) and 10 (9.3-10.7) months, respectively, whereas in the validation group, they were 10 (8.9-11.1) and 11 (9.6-12.4) months.

**Table 1:** Characteristics of patients with MPM in the training and validation group.

Characteristics	Total (n = 1978)	Training group (n = 1385)	Validation group (n = 593)	*P value*
	no.(%)	no.(%)	no.(%)
**Age**				0.997
<65	524 (26.5%)	363 (26.2%)	161 (27.2%)	
65-75	713 (36.0%)	497 (35.9%)	216 (36.4%)	
76-85	558 (28.2%)	395 (28.5%)	163 (27.5%)	
>85	183 (9.3%)	130 (9.4%)	53 (8.9%)	
**Gender**				0.905
Male	1545 (78.1%)	1085 (78.3%)	460 (77.6%)	
Female	433 (21.9%)	300 (21.7%)	133 (22.4%)	
**Race**				0.936
White	1785 (90.2%)	1258 (90.8%)	527 (88.9%)	
Black	101 (5.1%)	69 (5.0%)	32 (5.4%)	
Others	92 (4.7%)	58 (4.2%)	34 (5.7%)	
**Insurance**				0.897
Yes	1472 (74.4%)	1034(74.7%)	438(73.9%)	
No/Unknown	506 (25.6%)	351 (25.3%)	155 (26.1%)	
**Marital status**				0.976
Married	1301 (65.8%)	912 (65.8%)	389 (65.6%)	
Others	677 (34.2%)	473 (34.2%)	204 (34.4%)	
**Histology**				0.979
NOS	636 (32.2%)	451 (32.6%)	185 (31.2%)	
Sarcomatoid	249 (12.6%)	166 (12.0%)	83 (14.0%)	
Epithelioid	875 (44.2%)	615 (44.4%)	260(43.8%)	
Biphasic	218 (11.0%)	153 (11.0%)	65 (11.0%)	
**Grade**				0.853
I-II	46 (2.3%)	32 (2.3%)	14 (2.4%)	
III-IV	153 (7.6%)	98 (7.1%)	55 (9.3%)	
Unknown	1779 (89.9%)	1255 (90.6%)	524 (88.3%)	
**Site**				0.963
Left	760 (38.5%)	525 (37.9%)	235 (39.6%)	
Right	1166 (58.9%)	824 (59.5%)	342 (57.7%)	
Bilateral	52 (2.6%)	36 (2.6%)	16 (2.7%)	
**T stage**				0.966
T1	628 (31.7%)	449 (32.4%)	179 (30.2%)	
T2	447 (22.6%)	318 (23.0%)	129 (21.8%)	
T3	348 (17.6%)	235 (17.0%)	113 (19.1%)	
T4	555 (28.1%)	383 (27.6%)	172 (29.0%)	
**N stage**				0.988
N0	1285 (65.0%)	904 (65.3%)	381 (64.2%)	
N1	99 (5.0%)	68 (4.9%)	31 (5.2%)	
N2	540 (27.3%)	374 (27.0%)	166 (28.1%)	
N3	54 (2.7%)	39 (2.8%)	15 (2.5%)	
**M stage**				0.642
M0	1643 (83.1%)	1161 (83.8%)	482 (81.3%)	
M1	335 (16.9%)	224 (16.2%)	111 (18.7%)	
AJCC stage				0.931
I	446 (22.5%)	324 (23.4%)	122 (20.6%)	
II	282 (14.3%)	204 (14.7%)	78 (13.2%)	
III	508 (25.7%)	353 (25.5%)	155 (26.1%)	
IV	742 (37.5%)	504 (36.4%)	238 (40.1%)	
**Surgery**				0.597
Yes	626 (31.6%)	424 (30.6%)	202 (34.1%)	
No/Unknown	1352 (68.4%)	961 (69.4%)	391 (65.9%)	
**Chemotherapy**				0.977
Yes	1167 (59.0%)	818 (59.1%)	349 (58.9%)	
No/Unknown	811 (41.0%)	567 (40.9%)	244 (41.1%)	
**Radiation**				0.326
Yes	268 (13.5%)	167 (12.1%)	101 (17.0%)	
No	1710 (86.5%)	1218 (87.9%)	492 (83.0%)	

MPM, malignant pleural mesothelioma; NOS, not otherwise specified; AJCC, American Joint Committee on Cancer.

### Independent prognostic factors selection and nomogram construction

**Figure 2:**
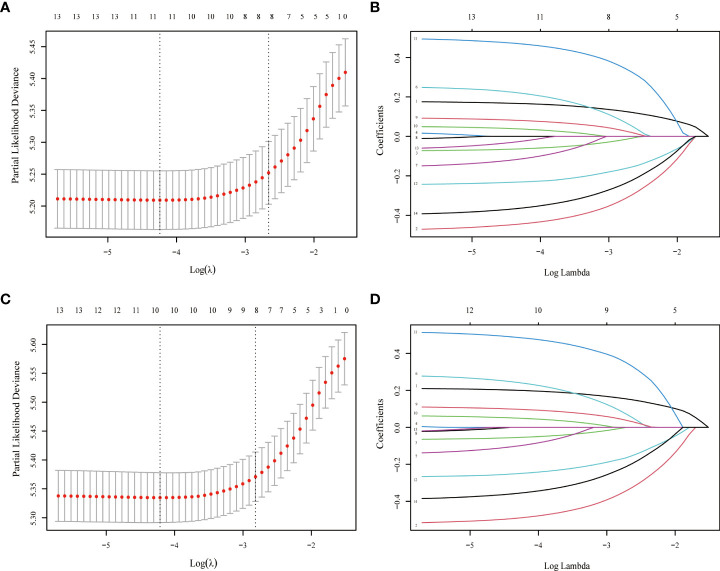
LASSO regression model was used to select characteristic impact factors for OS and CSS. Selection of tuning parameter (λ) for the LASSO model in OS **(A)** and CSS **(C)** LASSO coefficients of fourteen features in OS **(B)** and CSS **(D)**.

**Figure 3:**
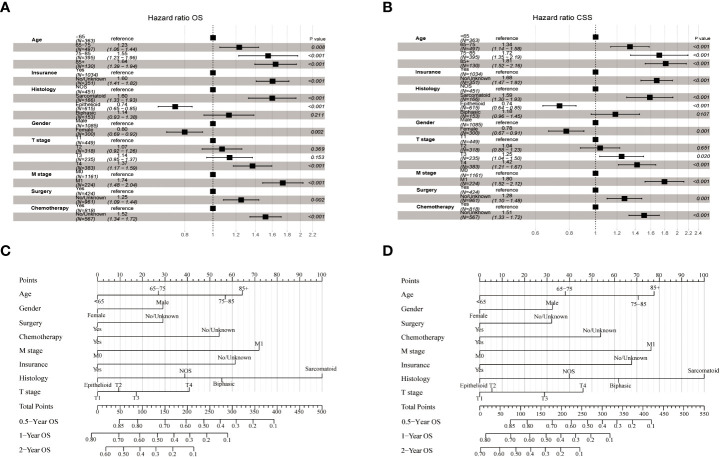
Forest plot demonstrating the LASSO-Cox regression model for OS **(A)** and CSS **(B)** in the training cohort and the Nomogram for predicting 0.5-, 1- and 2-year OS **(C)** and CSS **(D)**.

The LASSO-Cox regression analysis was used to determine the optimal coefficient for each prognostic factor based on the smallest partial probability deviation and generate coefficient curves from logarithmic (lambda) series (**Figure 2**). Age, gender, histology, insurance, T-stage, M-stage, surgery, and chemotherapy were found to be the eight independent predictors in the OS and CSS models, according to the minimum requirements for Lasso-cox regression analysis utilizing 10-way cross-validation (**Figure 2**). Then, to create nomogram-based survival prediction models for OS and CSS, respectively, we integrated these chosen candidate factors into a multivariate Cox regression model (**Figure 3**). The findings revealed that the strongest association between prognosis and histological type was found, with M stage, age, insurance, chemotherapy, T stage, surgery, and gender following. The scores for the chosen variables can be used to quickly and logically compute the survival odds for specific patients. **Figure 3** displays the results of the variables in the Nomogram.

### Validation of the nomogram

**Figure 4:**
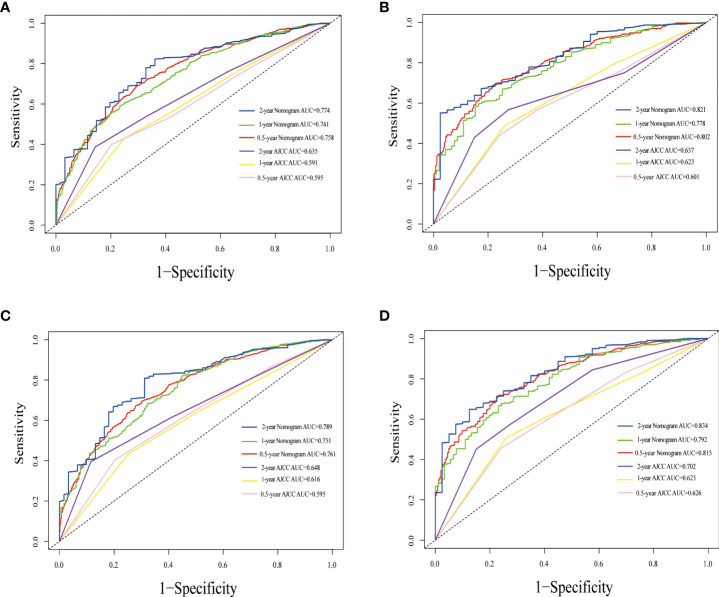
Time-dependent ROC curves comparing the use of the Nomogram and AJCC TNM staging system to predict the 0.5-, 1- and 2-year OS and CSS in the training cohort **(A, C)**, the internal validation cohort **(B, D)**.

The nomogram estimating overall survival at 0.5-, 1- and 2-year showed good predictive power, with a C-index of 0.729 for the training cohort and 0.717 for the validation cohort, which were all higher than that of the TNM staging system (training cohort: 0.611; validation cohort: 0.595). Likewise, the 0.5-, 1- and 2-year CSS accuracy for model prediction was also significantly better than the TNM staging system, with C-indexes of 0.711 vs. 0.592 in the training cohort and 0.721 vs. 0.639 in the validation cohort, respectively. Compared with the TNM staging system, the 0.5-, 1-year, and 2-year AUCs of the nomograms were all greater than 0.7, suggesting that the models had significantly greater predictive power (**Figure 4**). The calibration plots demonstrated agreement between predicted and actual survival. The 0.5-, 1- and 2-year OS (**Supplementary Figure 1**) and CSS predictions (**Supplementary Figure 2**) made by the nomogram models for the training and validation cohort showed good accuracy. With a wide range of favorable threshold probabilities, nomogram-based OS or CSS model decision curve analysis demonstrated high clinical value and predictive efficiency in predicting 0.5-, 1-year, and 2-year survival (**Supplementary Figures 3, 4**).

### Development of an dynamic online survival estimate calculator

**Figure 5:**
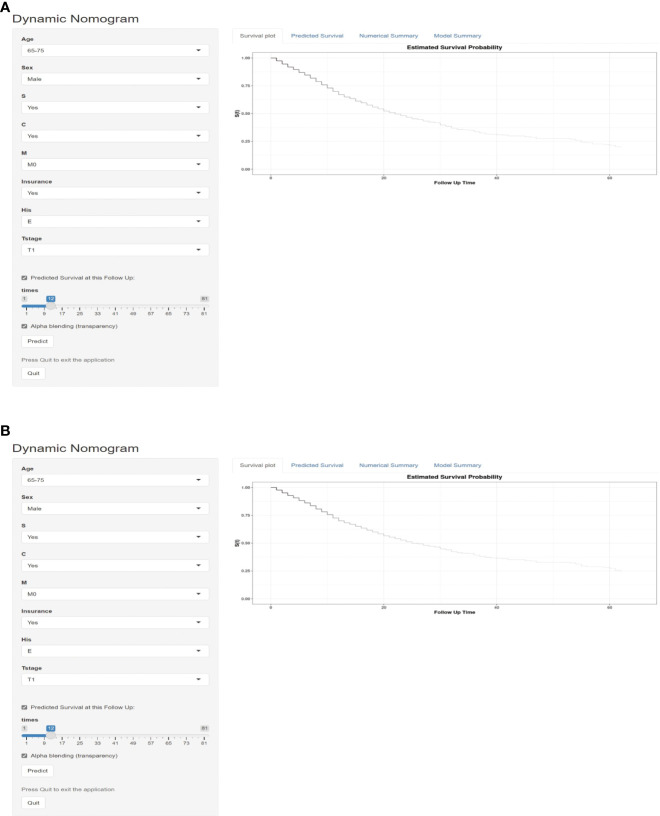
Dynamic survival risk calculator for OS **(A)** and CSS **(B)** in a MPM patient aged 65-75 years, male with insured, T1, M0, Epithelioid disease,with surgery and chemotherapy.

For further use by researchers and physicians, an online version of our nomograms for OS and CSS in MPM patients is available at https://mpmsurvival.shinyapps.io/MPMforOS/ and https://mpmsurvival.shinyapps.io/MPMforCSS/ (**Figure 5**). It is simple to calculate the estimated survival probability over time by entering clinical features and examining the figures and tables that the web server outputs. For example, the 1-year OS and CSS rate were approximately 67.0% (95% CI 62.0-72.0%) and 70.0% (95% CI 65.0-75.0%) for patients aged 65-75 years, male with insured, T1, M0, Epithelioid disease,with surgery and chemotherapy.

### Risk stratification based on the nomogram

**Figure 6:**
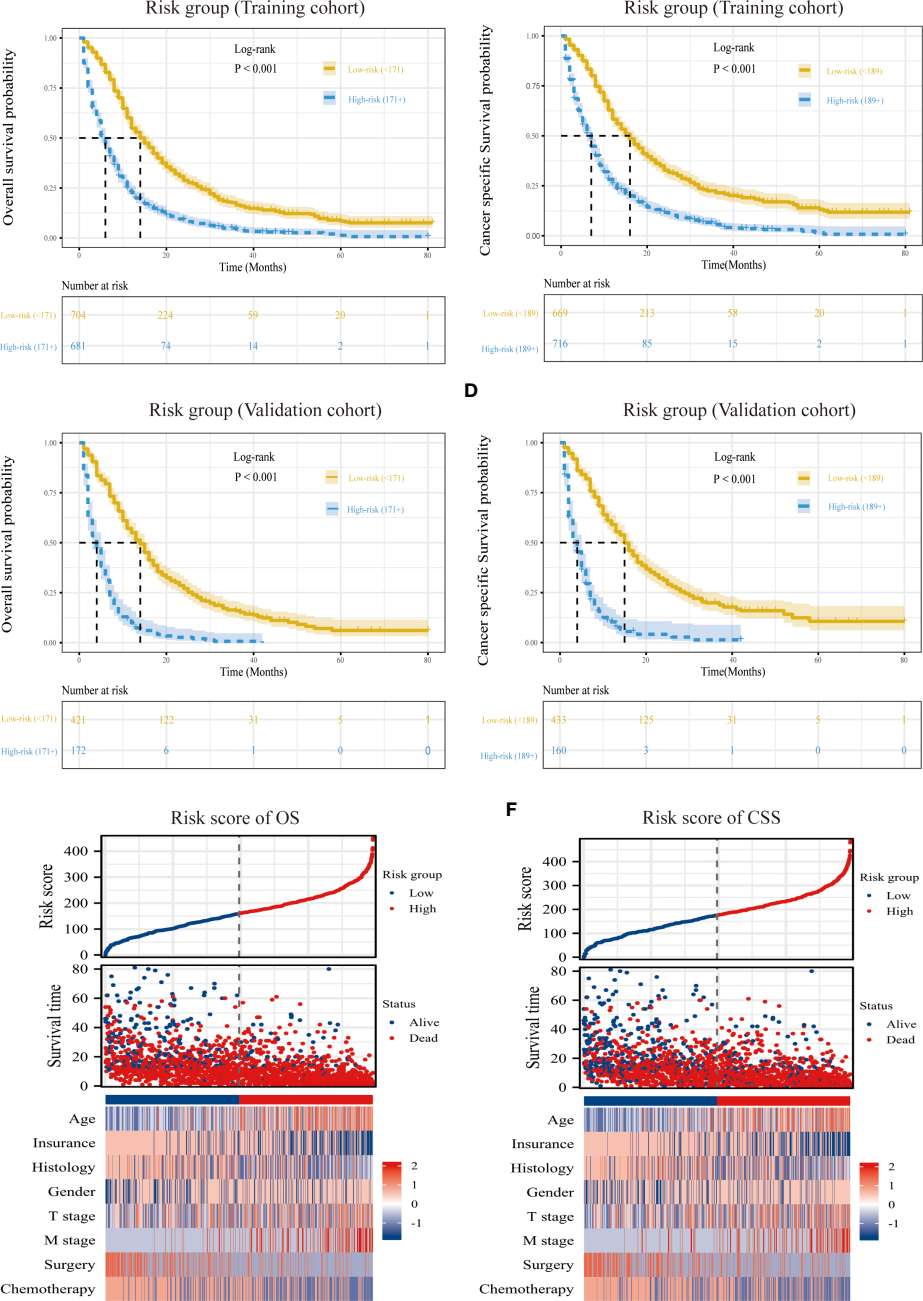
Overview of risk-stratification system according to risk points calculated by nomogram. OS and CSS analysis of patients with MPM in the training cohort **(A, C)** and validation cohort **(B, D)**. The distribution of clinicopathological features in different risk groups for OS **(E)** and CSS **(F)**.

According to the ideal cutoff point determined by the Survminer software program, patients classified according to the nomogram were split into high-risk and low-risk groups (OS model: 171 points; CSS model: 189 points). The survival curves of patients in different risk groups in OS and CSS were significantly different (P<0.001), which provided preliminary evidence of the value of the nomogram and the risk stratification system (**Figures 6A–D**). The distribution of abnormal clinical characteristics by OS (**Figure 6E**) and CSS (**Figure 6F**) risk groups was also displayed using heatmaps associated with risk factors.

### Radiotherapy selection based on different risk groups

We also looked at how crucial radiotherapy is for patients in various risk groups. According to the findings, radiation therapy patients in the low-risk group outlived those who did not receive it by a significant survival advantage (Median OS: 18 vs. 14 months, P=0.017; Median CSS: 18 vs. 15 months, P=0.037; **Supplementary Figure 5**). In contrast, radiation had no effect on OS or CSS in the high-risk group, and there was no statistically significant difference between the two groups (**Supplementary Figure 5**). The survival improvement of radiation in low-risk patients needs further verification, nevertheless, because there are so few people undergoing it.

## Discussion

A rare kind of cancer with a terrible prognosis, MPM is extremely aggressive. According to studies, the disease has an incubation period of up to 40 years and is primarily linked to asbestos exposure (3). Half a century ago, the incidence of MPM was significantly higher in developed countries than in developing countries due to the rapid industrialization process (22, 23). In recent years, the incidence of MPM in developing countries such as China has been on the rise, and the scenario is not encouraging (24). Because MPM develops slowly, has a difficult time being diagnosed, and progresses quickly, most patients with it get demoralizing treatment and results (25). A tailored and accurate survival risk evaluation system that can predict patient outcomes and help with medical decision-making is currently urgently needed in the clinical setting to increase patient survival.

One of the most reliable source of statistics on cancer incidence, diagnosis, treatment, and survival in the United States is the SEER database (26). The database compiles and disseminates information on cancer incidence and survival from 18 cancer registries, which accounts for around 35% of the country’s population. And it has received a lot of attention in the field of cancer research and offers outstanding advantages for the examination of uncommon tumors (27–29). In this study, we recruited a large cohort of patients diagnosed with MPM from SEER (n=1978) and identified eight clinical features associated with prognosis (age, gender, histology, insurance, T stage, M stage, surgery, chemotherapy). These features are good predictors of survival in MPM based on training and validation sets. This is, to the best of our knowledge, the first big SEER-based study to establish a nomogram predictive model for MPM overall survival and cancer-specific survival, excluding the TNM staging method. In addition, a dynamic web-based survival risk calculator was utilized to increase the clinical applicability of predictive models.

Epithelioid, biphasic, and sarcomatoid are the three main histological subtypes of MPM, a heterogeneous collection of tumors (30, 31). According to studies, sarcomatoid has the worst prognosis while epithelium is the most prevalent kind and has best prognosis (32). The findings of this research also demonstrated that the histological type is a distinct risk factor for prognosis, with the sarcomatoid type having the highest risk score in this model (100 points). One of the major contributing factors to a poor prognosis for patients with different malignancies is advanced age (33, 34). This study demonstrates that the majority of newly diagnosed patients are old (age>65, n=1454, 73.5%), and the likelihood of a bad prognosis rises with age. This may be due to the disease’s exceptionally long incubation time (35). Gender was found to be an independent risk factor in the current investigation, with female patients having a lower incidence (n=433, 21.9%) and better survival rates than male patients. This difference may be due to the fact that more men than women work in the asbestos business. The prognosis of cancer patients is strongly correlated with insurance status, according to a number of studies (36–38). According to this study, people with insurance had higher OS and CSS than patients without insurance. TNM staging system is still imperfect but is currently used routinely to direct the care of cancer patients (15–18). Prior research has suggested that the size of the primary tumor is also an important factor affecting the prognosis of patients with MPM (16). Our research demonstrates that, in the 7th TNM staging system, regional lymph node metastasis (N stage) is less accurate in predicting the prognosis of MPM patients than initial tumor invasion (T stage) and distant metastasis (M stage), and the majority of patients are in N0 stage(n=1285, 65.0%). This may be due to the N staging of MPM being borrowed from the N staging of non-small cell lung cancer, which is not always appropriate to extrapulmonary tumors (15).

The current study goes into more detail about the connection between medical interventions and patient outcomes, demonstrating that chemotherapy and surgery are still crucial for improving patient survival. Patients with resectable MPM typically elect surgery. Pleurectomy/decortication (P/D) and extrapleural pneumonectomy (EPP) are definitive procedures, and patients benefit greatly from postoperative adjuvant chemotherapy. Since only 20% of patients with early MPM can undergo surgery, the pemetrexed plus cisplatin chemotherapy regimen is used as the first option for unresectable patients (39–41). Prior to research, MPM was believed to be radiation therapy resistant, but studies now demonstrate that radiation therapy can have beneficial therapeutic effects12. Recent clinical investigations have demonstrated that the median survival of patients undergoing high-dose chemoradiotherapy following EPP is 23.9–39.4 months due to the widespread use of intensity-modulated radiation therapy (IMRT) (42). The choice of the ideal radiation timing, however, is still up for debate. Considering the variability of prognosis of patients with different disease degrees, this study classified patients into high-risk and low-risk groups according to risk score and subsequently investigated the clinical importance of radiation in different risk groups. The findings indicate that radiotherapy significantly improves survival for patients in the low-risk group but not for those in the high-risk group, which may serve as a foundation for the therapeutic application of radiotherapy in MPM patients. It should be noted that due to the lack of important information such as radiation dose and target volume in the SEER database, and the small sample size of radiotherapy patients in this cohort study, the above conclusions need further verification.

In this work, we used LASSO-Cox regression analysis to add eight prognostic features into our final survival risk evaluation model. The multidimensional validation also revealed that the model had great accuracy and significantly exceeded the traditional TNM staging approach. The choice of these parameters was logical, practical, and feasible. The current study has limitations, which must be acknowledged. Because this was a retrospective analysis, patients who were missing from the SEER database were not included, which could have resulted in sampling error. Additionally, a few crucial clinical information, such as performance score, radiation dose, target area, details of chemotherapy (cycles and regimens), and second line therapy are missing in the SEER registry, which may have reduced the model’s long-term prognostic potential. Third, there may be some collinearity among the variables included in the model, which may lead to overfitting in the model. Finally, further experimental data will be needed to confirm the validity and applicability of the nomogram developed in this study.

## Conclusions

In conclusion, through a detailed analysis of patient records in the SEER database between 2010-2015, we found that multiple clinical factors have an independent impact on the OS and CSS in patients with MPM, and successfully constructed and validated predictive models with good accuracy. With the aid of this model, patients and doctors will be better able to comprehend the prognosis.

## Data availability statement

The original contributions presented in the study are included in the article/**Supplementary Material**. Further inquiries can be directed to the corresponding author.

## Author contributions

ML had full access to all of the data in the study and takes responsibility for the integrity of the data and the accuracy of the data analysis. Concept and design: SC, WY, ML. Acquisition, analysis, or interpretation of data: SC, WY, SS, JX, HB, YP. Drafting of the manuscript: SC, WY, ML. Critical revision of the manuscript for important intellectual content: ML. Statistical analysis: SC, WY. Obtained funding: ML. Administrative, technical, or material support: ML. Supervision: ML. All authors contributed to the article and approved the submitted version.

## Funding

The study was supported by a grant from Army Medical University to ML and the Natural ScienceFoundation of Chongqing, China (No. Cstc2019jcyjjqX0008).

## Acknowledgments

We are immensely grateful to all investigators involved in this study.

## Conflict of interest

The authors declare that the research was conducted in the absence of any commercial or financial relationships that could be construed as a potential conflict of interest.

## Publisher’s note

All claims expressed in this article are solely those of the authors and do not necessarily represent those of their affiliated organizations, or those of the publisher, the editors and the reviewers. Any product that may be evaluated in this article, or claim that may be made by its manufacturer, is not guaranteed or endorsed by the publisher.
