# Supplementary material for: Establishment of predictive nomogram and web-based survival risk calculator for malignant pleural mesothelioma: A SEER database analysis
Source: Front Oncol. 2022 Oct 7;12:1027149. doi: 10.3389/fonc.2022.1027149 (PMC9585232; doi:10.3389/fonc.2022.1027149)
Supplement: Supplementary file 1 [file DataSheet_1.docx]

Supplementary Material


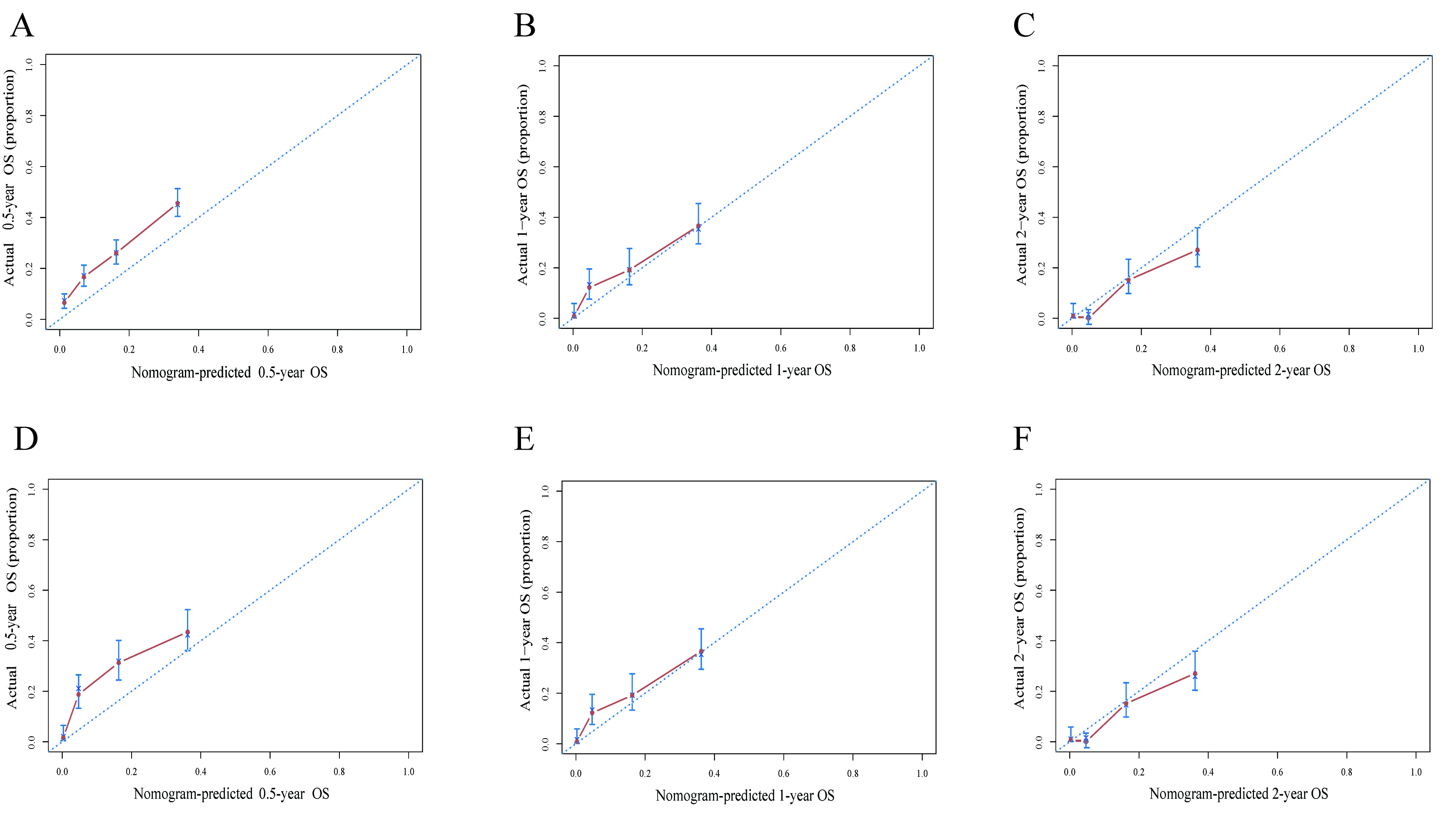


Supplementary Figure 1. The calibration curves predicting 0.5-year (A,D), 1-year (B,E), and 2-year (C,F) OS in training group and validation group.


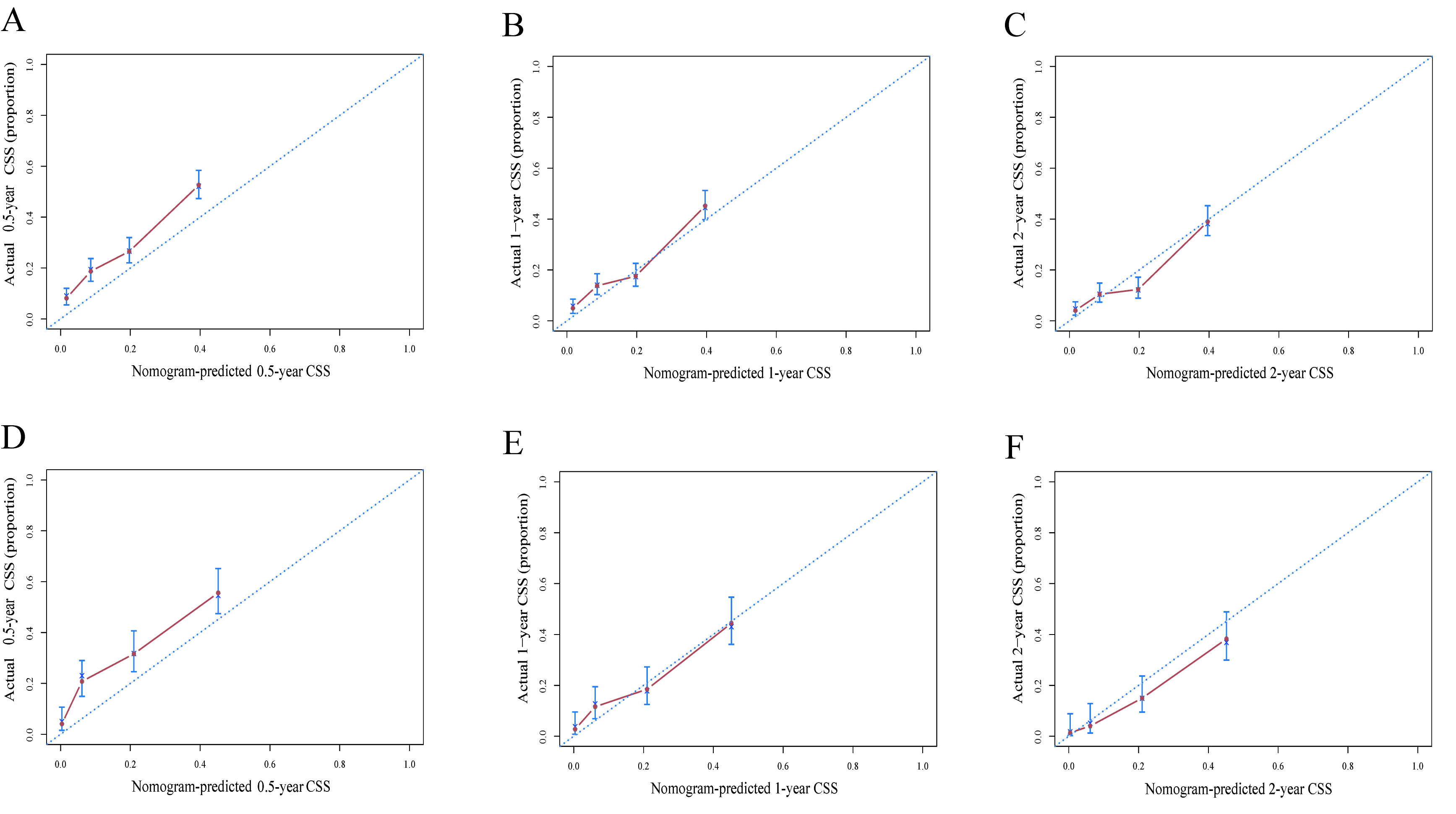


Supplementary Figure 2. The calibration curves predicting 0.5-year (A,D), 1-year (B,E), and 2-year (C,F) CSS in training group and validation group.


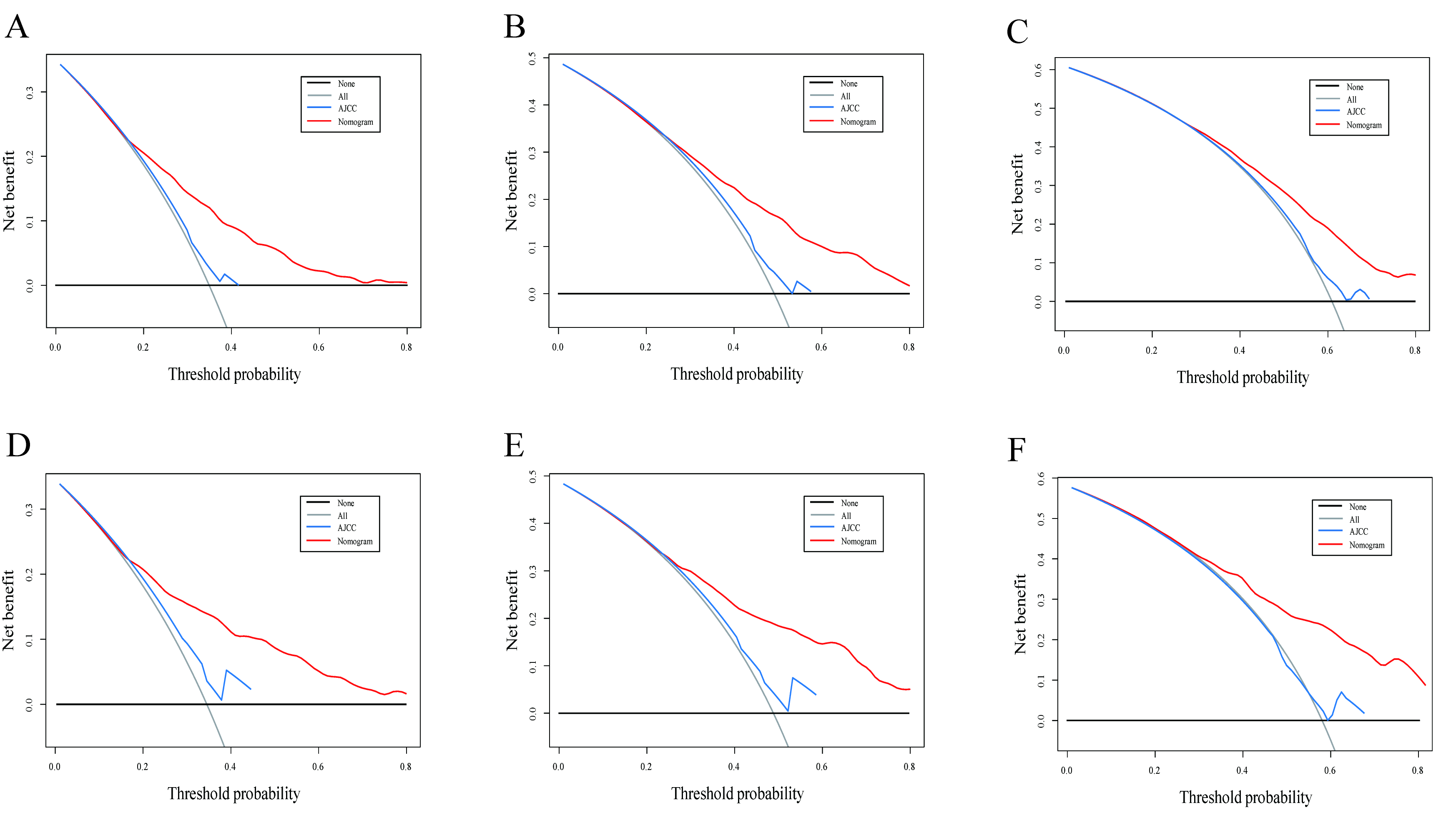


Supplementary Figure 3. The nomogram and the AJCC stage of the Decision curve analysis in the prediction of OS at the 0.5-year (A,D), 1-year (B,E) and 2-year (C,F) point in the training and validation groups.


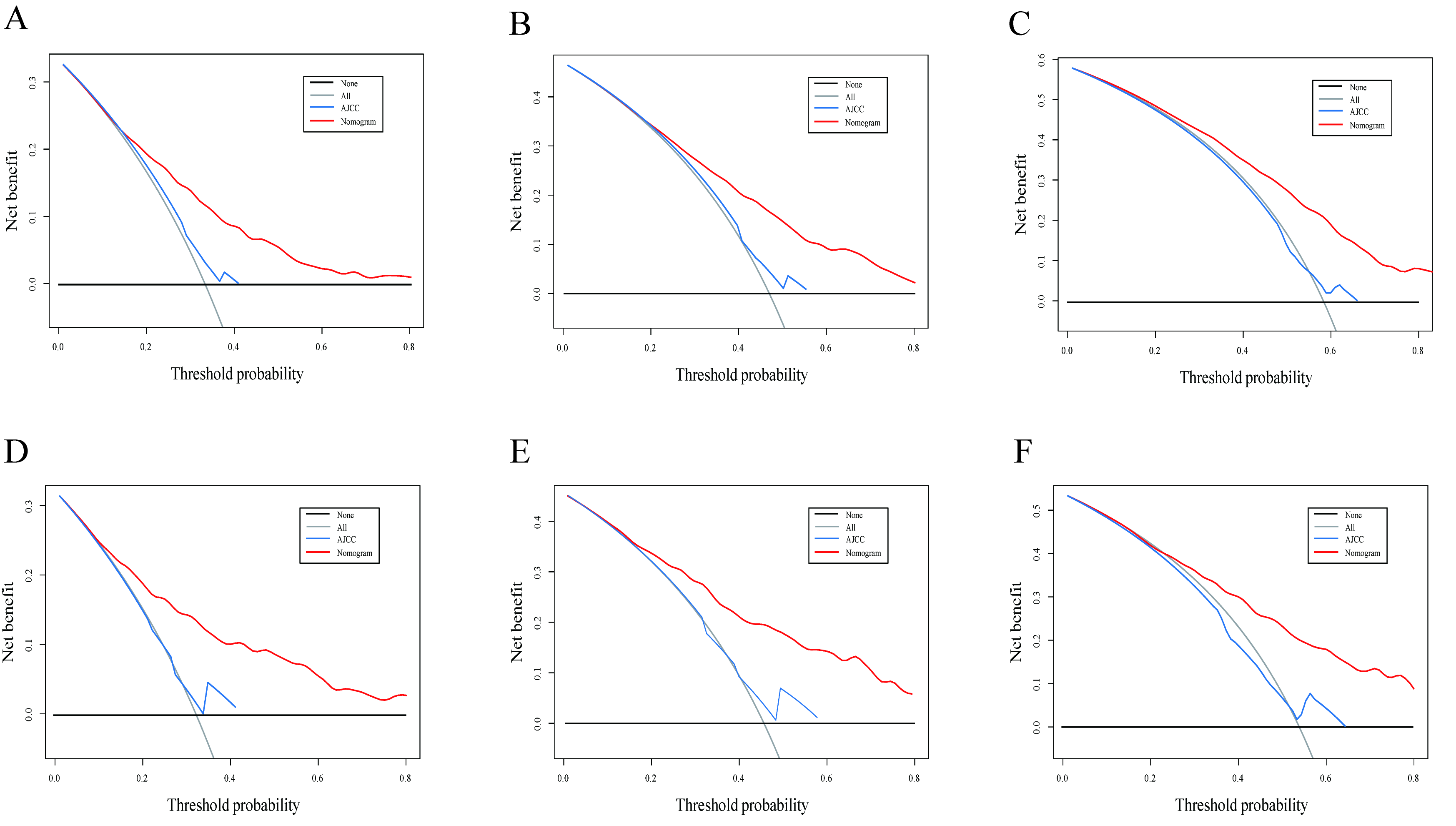


Supplementary Figure 4. The nomogram and the AJCC stage of the Decision curve analysis in the prediction of CSS at the 0.5-year (A,D), 1-year (B,E) and 2-year (C,F) point in the training and validation groups.


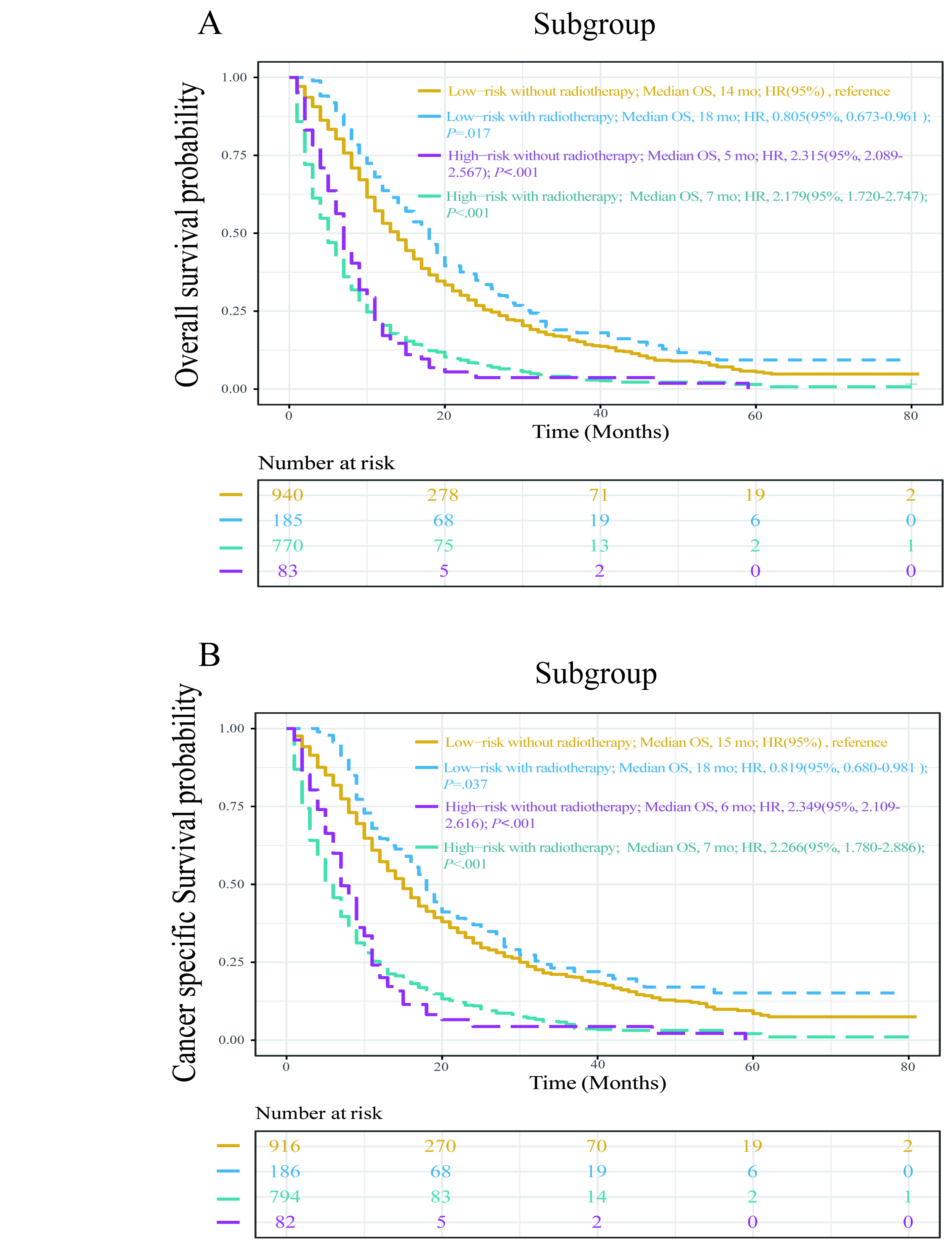


Supplementary Figure 5. Differences in OS (A) and CSS (B) by radiotherapy selection based on risk group.
